# Nephroprotective Impacts of *Hyphaene thebaica* (Doum) Against Gentamicin‐Induced Renal Toxicity Through Different Signaling Pathways

**DOI:** 10.1002/fsn3.70345

**Published:** 2025-05-27

**Authors:** Saed A. Althobaiti

**Affiliations:** ^1^ Department of Biology Turabah University College, Taif University Taif Saudi Arabia

**Keywords:** gentamycin, *Hyphaene thebaica*, inflammation‐associated markers, kidney toxicity, renal markers

## Abstract

*Hayphaene thebaica* ( *H. thebaica* ) is thought to be effective in treating several diseases and has possible anti‐toxic impacts. Gentamycin (GM) toxicity in internal organs is mostly induced by oxidative stress production. Therefore, the current study aimed to examine the protective effect of *H. thebaica* ) against gentamycin‐induced renal toxicity. Twenty‐four male mice were allocated into four groups: first group administered saline orally as a control, the second group administered *H. thebaica* extract (5%) for 2 weeks, third group injected intraperitoneally with GM (100 mg/kg, daily) for 15 days, and fourth group served as the protective one, receiving doses used in second and third groups for 2 weeks. To examine kidney function, serum was extracted. Renal tissues were collected and examined for gene expression, histopathology, and immunohistochemistry for nuclear factor kappa B (NF_k_B), transforming growth factor beta‐1 (TGF‐β1), and tumor necrosis factor‐alpha (TNF‐α) immunoreactivity. Serum levels of creatinine, uric acid, and urea were all increased after gentamicin injection. TGF‐β1 and cyclooxygenase‐2 (COX2) were upregulated in renal tissues of the gentamycin group compared to the control and *H. thebaica* groups using real‐time quantitative PCR (RT‐qPCR) analysis, unlike genes of oxidative stress markers, nuclear factor erythroid 2‐related factor 2 (Nrf2) was downregulated and was ameliorated by *H. thebaica* pre‐administration. Immunoreactivity of genes of TGF‐β1, NF_k_B, and TNF‐α was significantly immunoreacted and expressed in GM‐injected mice and was normalized and ameliorated in the protective group. There were deformities in renal histology represented by degenerative changes in the renal glomerulus, which was surrounded by a wide capsular space, degeneration in the tubular epithelium, and an increase in the accumulation of acidophilic proteinaceous substances within the lumens of some tubules in the gentamycin‐injected group compared to the protective group. These deformities were ameliorated by *Hyphaene thebaica* administration in the protective group.

## Graphical abstract

*Hyphaene thebaica* extract ameliorated nephrotoxic effects of gentamycin through its role as anti‐inflammatory mediators by regulating NFkB, COX‐2, and TGF‐β1 expressions. Moreover, *H. thebaica* regulated the expression of inflammation‐associated apoptotic genes. *H. thebaica* increased antioxidative stress biomarkers named Nrf2, catalase, and GSH. Therefore, using *H. thebaica* as a supplemental nutrient against toxicity of the kidney is acceptable with limitation to its possible impacts for use in medication.

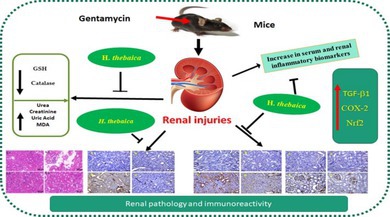

## 1 Introduction

Acute tubular necrosis, which is marked by severe renal impairment and requires renal replacement therapy, acute tubulointerstitial nephritis, different types of glomerulonephritis, and osmotic nephrosis are all examples of drug‐induced nephrotoxicity. The latter could be connected to the need for long‐term hemodialysis and the development of chronic nephropathy. The main medicines that induce medication‐associated nephrotoxicity, which affects 18%–27% of individuals, include calcineurin inhibitors, aminoglycosides, amphotericin B, and non‐steroidal anti‐inflammatory drugs (NSAIDs) (Petejova et al. 2020).

Gentamycin (GM) is an aminoglycoside antibiotic that is commonly used as a broad‐spectrum antibiotic. Reports suggested repeated use of gentamycin may develop organ dysfunction, especially renal impairments (Ali 1995). Several research studies showed that the toxicity of gentamicin is induced by the activation of different pathways of inflammation and the lowering of renal biomarkers (Al‐Johani and Al‐Sowayan 2023). Gentamycin toxicity includes cell destruction, congestion, and fibrosis of the kidney and upregulation in the expression of tumor necrosis (Erseçkin et al. 2022). The cell types where gentamicin accumulates are the ones that experience cytotoxicity. Gentamicin accumulation is greater in these cells, which is compatible with the development of a protein‐cation transporter (Fujiwara et al. 2009). Antioxidants and anti‐inflammatory biomarkers have been shown to decrease or even reverse gentamicin‐induced kidney damage (Erseçkin et al. 2022). When gentamicin is administered to experimental mice, tubular epithelial cells undergo necrosis and apoptosis (Li et al. 2009; Edwards et al. 2007).

Natural sources of medical background have existed for decades. About 65% to 80% of people worldwide, particularly in underdeveloped nations, prefer it as their major source of healthcare. This is because it is more culturally acceptable, more compatible with the human body, and has less adverse consequences (Amin Mohamed et al. 2010; Shady et al. 2021). Medicinal plants are common plants used in Saudi Arabia for the treatment of different forms of illness and organ toxicities. *Hayphaene thebaica* ( *H. thebaica* ), Doum, is a palm tree that is well‐known in Arabian nations and has edible oval fruit; it is said to be effective in treating hypertension. Diabetes, hematuria, hemorrhage, and bilharziasis can all be treated with the aqueous extract of *H. thebaica* , particularly after childbirth (Salib et al. 2013). Based on earlier published papers, *H. thebaica* lowers blood lipids and has antibacterial, antioxidant, and anti‐diabetic properties (Eldahshan et al. 2009; Shehu et al. 2017; Hassan 2020). Furthermore, in diabetic rats, *H. thebaica* extracts have the potential to lower blood glucose levels.

Therefore, the current study aimed to examine the protective impacts of *H. thebaica* extract against GM‐induced renal toxicity through the regulation of renal biomarkers, antioxidants, and apoptotic and inflammatory pathways at biochemical, genetic, and cellular levels.

## 2 Materials and Methods

### 2.1 Reagents and Chemicals

Chemicals and reagents used are gentamicin and normal saline (0.9%). The primary antibodies used are nuclear factor kappa‐b (NF‐κB), transforming growth factor beta‐1 (TGF‐β1) and tumor necrosis factor‐alpha (TNF‐α). Avidin‐biotin‐peroxidase complex ELISA kit, DAB (3,3‐diaminobenzidine), and biotinylated secondary antibody were from Santa Cruz, Philadelphia, USA. GM was purchased from Memphis Pharmaceutical Company, Cairo, Egypt.

### 2.2 Preparation of the *H. thebaica* Extract

After cleaning and removing any debris, the fruit was divided into seed and pulp. After drying, the pulp was powdered. Five hundred grams of the *H. thebaica* powder were macerated on ice. The extraction of Doum was done for 3 days, at a room temperature of 250°C–30°C in a Soxhlet extractor; distilled water 100% (v/v) was used as the solvent. A total of 0.05 mg/mL was obtained using the maceration method (Sankeshwari et al. 2018). Mice in the current study were given 5% *H. thabica* (5 mL) of the extract; 0.25 mg/5 mL was dissolved in 100 mL of regular water as directed, and they were freshly rehydrated every 3 days.

### 2.3 Animal Experiments

Twenty‐four male mice, 6 weeks of age, weighing 20g–30 g, were used for the current study. The animals were maintained at our labs and received free access to food and water. All procedures used in this experiment were in accordance with those established by the deanship of scientific research of Taif University. Seven days before the onset of the experiments, mice were handled to ensure adaptation. The mice were allocated into four groups randomly after coming to the experimental unit of our university. The first group received normal saline as a control for 2 weeks orally. The second group received 5% doum extract daily for 2 weeks orally (Yusuf et al. 2022). The third group was injected intraperitoneally with gentamicin at a dose of 100 mg/kg daily for 2 weeks (Althobaiti et al. 2024; Althobaiti 2024). The protective fourt group was injected with gentamicin intraperitoneally at a dose of 100 mg/kg daily, and 5% doum extract orally for 2 weeks. The dose of 5% doum extract (0.25 mg/5 mL) was almost 1.5 mL−1.4 mL.

### 2.4 Sampling

Following euthanasia, mice were subjected to decapitation after 2 weeks. Blood was taken from the retro‐orbital venous plexuses of the eyes. Blood was kept for clotting, then centrifuged at 2000 *xg* for 8 min to get serum from the supernatant layer. The extracted serum was kept at −20°C for future biochemical assays. Both kidneys from each mouse were taken; one part, in cold PBS, was homogenized, then centrifuged for 8 min at 2000 xg at 4°C. The clean supernatant layer was taken and preserved at −20°C for antioxidant measurements. Some parts of the kidney were soaked in (10%) formalin for histology and immunohistochemistry examination. The last part of the kidney was immersed in Qiazol for real‐time quantitative‐PCR analysis.

### 2.5 Kidney Biomarkers Measurements

Commercial Kits for urea, creatinine, and uric acid were purchased from a local company (*Biodiagnostic* company, Giza. Egypt) and were assayed following the protocols provided with each kit.

### 2.6 Antioxidants and Oxidative Stress Biomarkers Measurements in Renal Homogenate

Malondialdehyde (MDA), catalase (CAT) and reduced glutathione (GSH) of renal tissue homogenates were measured as stated before.

Catalase (CAT) activity was measured according to the protocol demonstrated by Luck (1965). Briefly, two buffer solutions were used, with the first comprising KH_2_PO_4_ (50 mM) at pH 7.0 and the second containing H_2_O_2_ (12 mM) in KH_2_PO_4_ (50 mM). In separate cuvettes, buffer solution 1 (900 μL) and buffer solution 2 (900 μL) were added, followed by the addition of 100 μL of the sample. After incubating the cuvettes in the dark for 45s–60 s, the absorbance was measured at 240 nm. CAT activity was determined by calculating the difference in absorbance after 45 and 60 s.

To measure reduced glutathione activity (GSH), the procedures of Rao et al. (1996) and Israr et al. (2006) were followed to measure GSH. Samples were added to a reaction mixture of 1 mL containing KH_2_PO_4_ buffer (100 mM) at pH 7.5, EDTA (1 mM), and oxidized glutathione (0.5 mM), followed by the addition of NADPH (0.2 mM) to initiate the reaction. The reaction was then allowed to proceed for 3 min, and absorbance was recorded at 340 nm using a UV–Vis spectrophotometer. The oxidative stress biomarker named malondialdehyde (MDA) was quantified in accordance with the methodology previously described (Mihara and Uchiyama 1978). The values were expressed as nmol/g tissue.

### 2.7 ***Quantification and Expression of TGF‐β1, COX2, Nrf2***, ***and SGLT2 Using RT‐qPCR Analysis***

RNeasy Mini kit (Qiagen, Valencia, CA, USA) was used to extract total RNA from renal tissues. The reverse transcription of extracted RNA was used to synthesize cDNA using the Quanti‐Tect Reverse Transcription Kit (Qiagen, 19,300 Germantown Road.

Germantown, MD, USA). cDNA was amplified using the SYBR Green Master Mix (Thermo Fisher Scientific, Waltham, MA, USA). Amplification and analysis of TGF‐β1, COX2, and Nrf2 expressions were performed using RT‐qPCR in the kidney, and beta‐actin was used as an internal standard and/or reference. The primer details were shown in Table 1. The results were quantified and calculated using the comparative cycle threshold (Ct) method and reported as relative fold change compared to the control gene expression. Primers were designed using Real‐time PCR (TaqMan) Primer and Probes Design Tool (https://www.genscript.com/tools/real‐time‐pcr‐taqman‐primer‐design‐tool).

**TABLE 1:** Primer sequence and real‐time quantitative PCR (RT‐qPCR) conditions of examined genes.

Gene	Accession number	Forward primer sequence (5′ → 3′)	Reverse primer sequence (5′ → 3′)	Amplicon size (bp)
TGF‐β1	NM_001419560	AGATCCCGCCAGAGTGCG	GACCAACAGACAAAAGCCCC	222
COX2	NM 017232	TGATCTACCCTCCCCACGTC	ACACACTCTGTTGTGCTCCC	190
Nrf2	NM_031789.2	TGACCATGAGTCGCTTGCC	TCCTGCCAAACTTGCTCCAT	153
β‐Actin	NM_031144.3	AGGAGGAGGAGGAGGAGGAG	TGGGAGGAGGAGGAGGAGAG	140

### 2.8 Histological and Immunohistopathological Results

Samples from the kidney were immersed in neutral formaldehyde solution (10%, Sigma‐Aldrich, USA) for 24 h. As described by Bancroft and Layton (2013), using a paraffin integration device renal tissues were fixed. Immunoreactivity of the kidney was noted before (Malkiewicz et al. 2006). The renal tissues were soaked in paraffin to dry, then sectioned, mounted, and incubated for 12 h. Renal tissue sections were then blocked for 30 min, then at 4°C, sections were incubated for 10 h with diluted polyclonal‐anti‐TGF‐β1, NF‐kB, and TNF‐α antibodies (1:1500). Next sections were washed with PBS for three times. Renal sections were incubated with biotinylated anti‐rabbit IgG (1:300 dilution) as a secondary antibody. Samples were finally incubated for 40 min at room temperature with avidin‐biotin‐peroxidase. As a counterstain for the samples, hematoxylin was used. DAB was added to visualize the peroxidase response.

### 2.9 Statistical Analysis

Data is presented as means ± SE. GraphPad Prism 5 program was used to analyze the data. One‐way ANOVA and Tukey–Kramer post‐analysis tests were used to analyze the present data. The significance was established at *p < 0.05, **p < 0.001, and ****p < 0.0001 between groups.

## 3 Results

### 3.1 Ameliorative Impacts of *H. thebaica* Against GM‐Induced Changes on Renal Biochemical Markers

**FIGURE 1:**
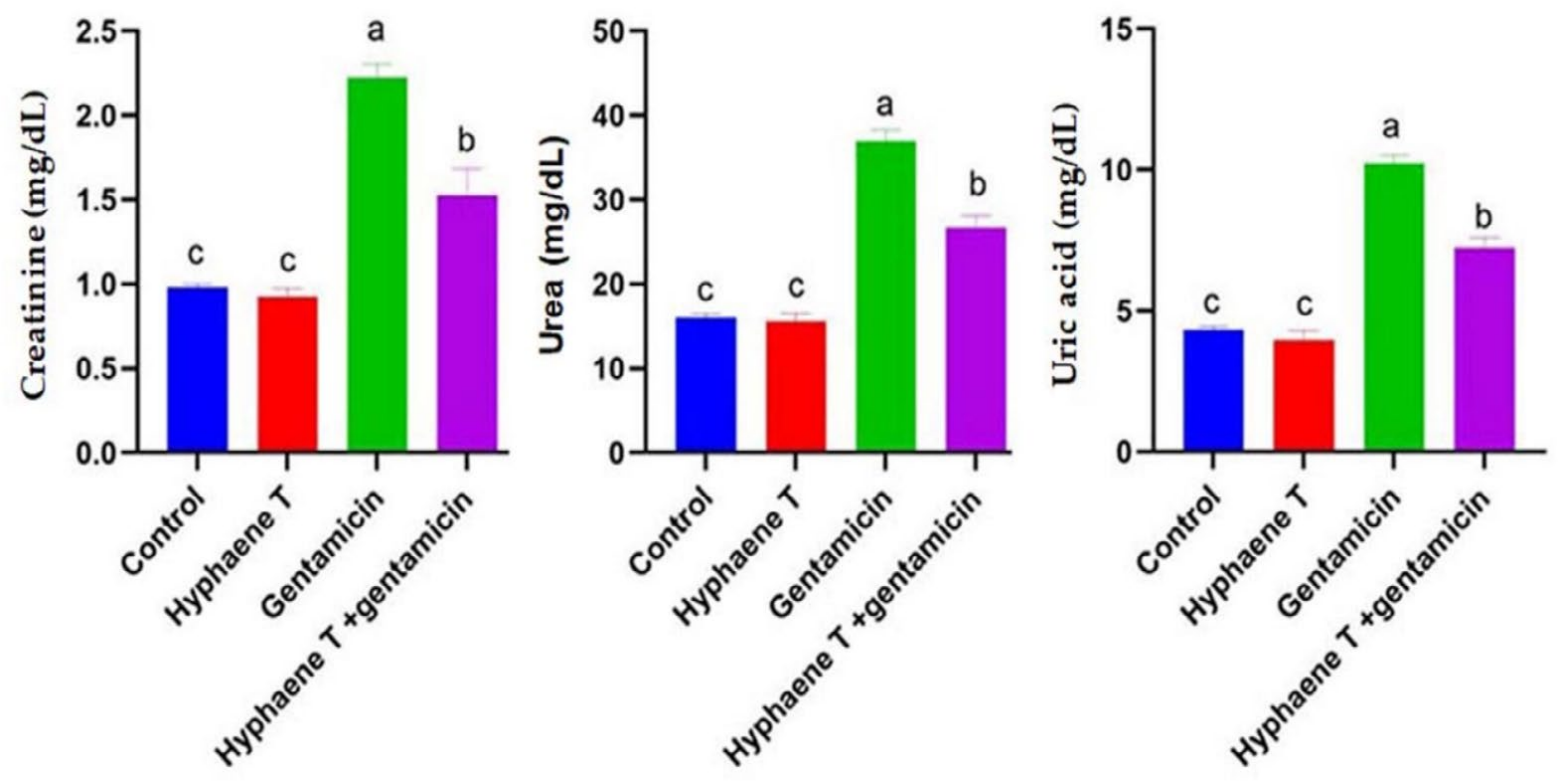
The effect of *Hyphaene thebaica* supplementation on the levels of creatinine, uric acid, and urea of different groups against gentamycin‐induced renal toxicity. Values are represented as mean ± SEM. Columns with different letters mean significant differences at *p* < 0.05.

Results in Figure 1 clarified that there was a significant increase in renal biomarkers (creatinine, uric acid, and urea) in GM‐injected mice compared to other groups. *H. thebaica* co‐administration with gentamycin significantly normalized and recovered the increase in biomarkers reported in GM‐injected mice.

### 3.2 Ameliorative Impacts of *H. thebaica* Against GM‐Induced Changes on Antioxidants and Oxidative Stress Biomarkers

**FIGURE 2:**
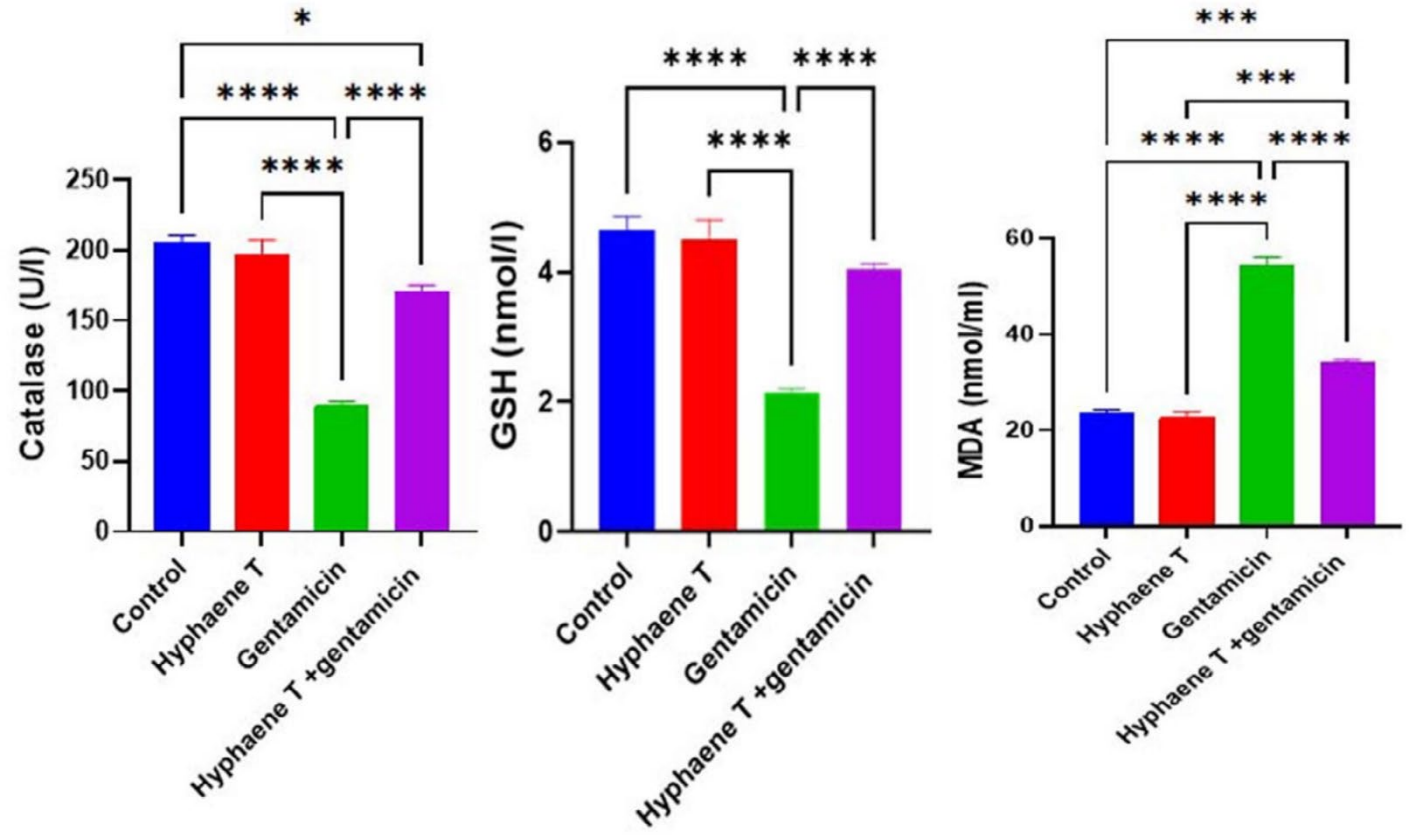
Impacts of *Hyphaene thebaica* supplementation on the level of catalase, GSH, and MDA in different groups after gentamycin‐induced renal toxicity. Values are represented as mean ± SEM. Columns with different sigma liters were significantly different. The significance levels were shown between bars at *p < 0.05, ***p < 0.001, and at ****p < 0.0001 between different groups as indicated on each separate figure.

The activity of antioxidant biomarkers such as catalase and GSH was significantly declined (*p* < 0.05) in GM‐injected mice compared to the control group. In contrast, renal contents of MDA were increased significantly in GM‐injected mice. In contrast, administration of *H. thebaica* revealed a significant reduction in MDA levels and a significant restoration in catalase and GSH activities compared to GM‐injected mice (Figure 2).

### 3.3 Ameliorative Impacts of *H. thebaica* Against GM‐Induced Changes in Apoptotic and Inflammatory Markers

**FIGURE 3:**
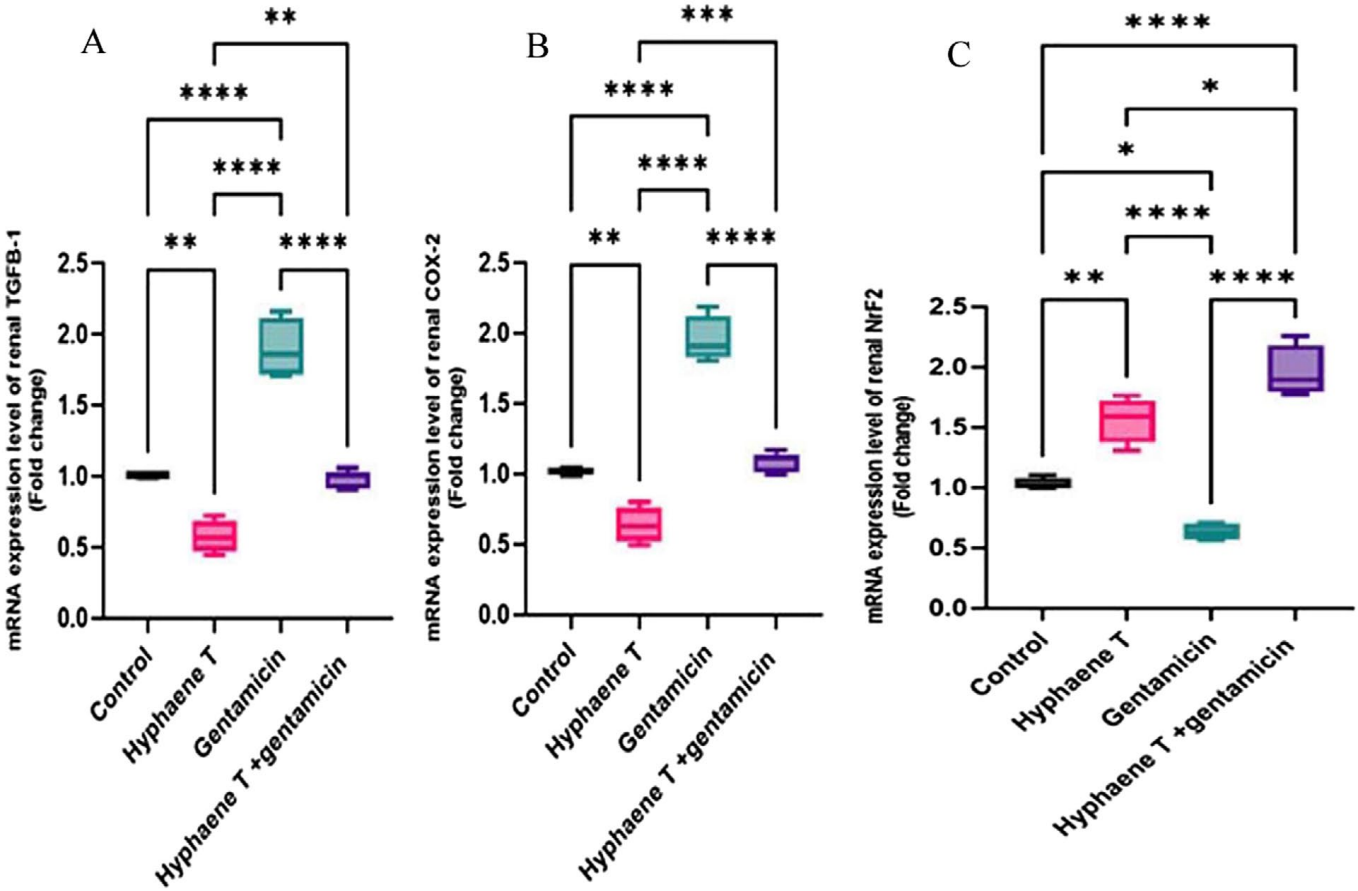
Quantification of mRNA expression of TGF‐β1 (A), COX2 (B), and Nrf2 (C) genes. The graphic presentations of examined genes were based on qRT‐PCR analysis for examined genes after normalization with β‐actin in different groups. The significance levels were shown between bars at *p < 0.05, **p < 0.01, ***p < 0.001, and ****p < 0.0001 between different groups as indicated on each separate figure.

Quantitative expression of inflammatory apoptotic markers (TGF‐β1 and COX2) showed significant upregulation in GM‐injected mice compared with control and *H. thebaica* groups. In parallel, the expression of antioxidative stress marker (Nrf2) was decreased in GM‐injected mice. TGF‐β1 and COX2 expression was down‐regulated significantly in the protective group compared to GM‐injected groups. On the other hand, there were significant restoration and upregulation in the Nrf2 expression protective group compared to GM‐injected mice **(**Figure 3).

### 3.4 Ameliorative Impacts of *H. thebaica* Treatment on GM‐Induced Alteration in Renal Histology

**FIGURE 4:**
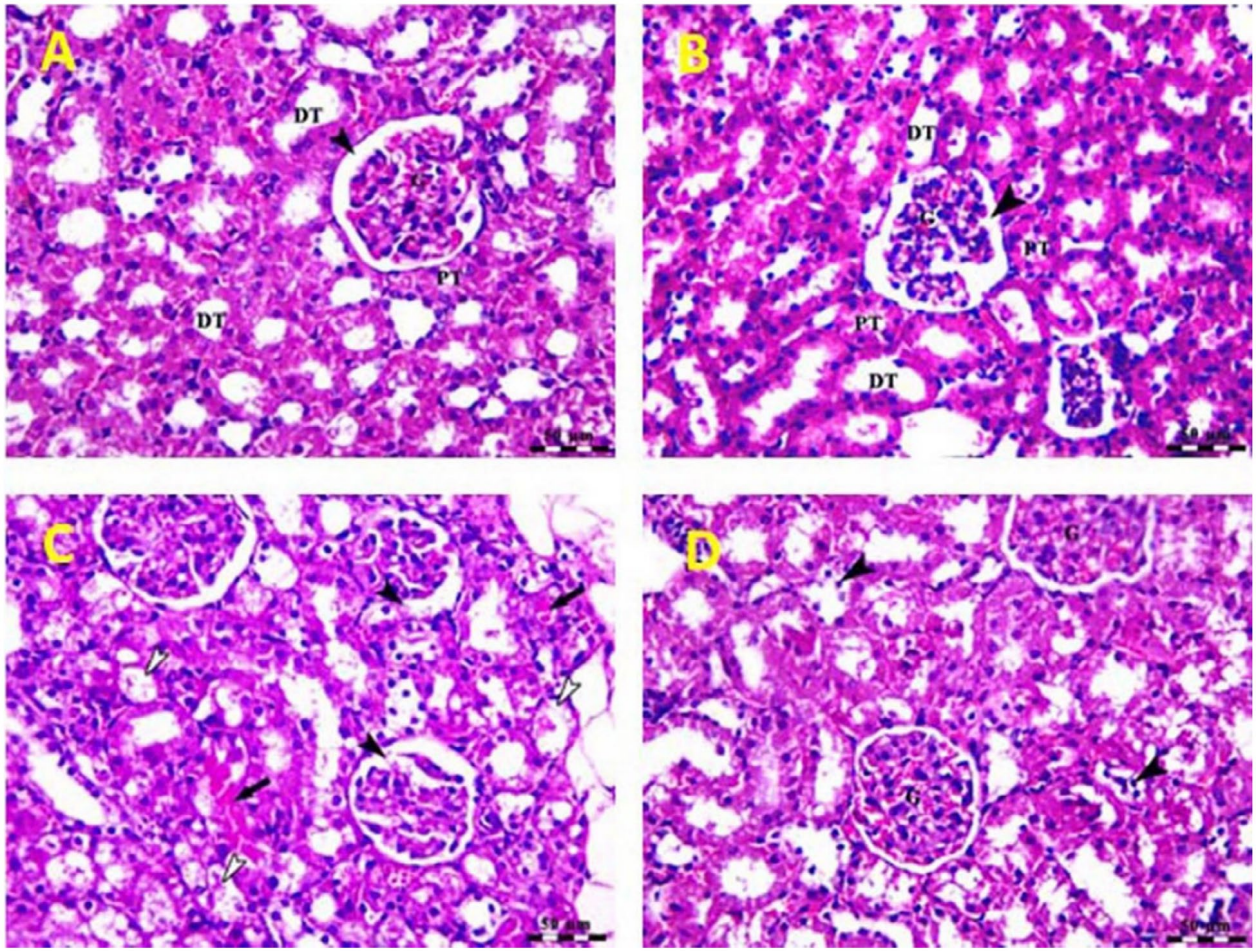
Photomicrograph of kidney of control and *Hyphaene thebaica* ‐treated group. (A, B) Showing normal structure of renal parenchyma with intact renal corpuscles containing glomeruli (G) which surrounded with capsular space (arrow heads) in addition to normal proximal (PT) and distal (DT) convoluted tubules. Stain H&E, Bar = 50 μm. (C) Photomicrograph of renal cortex of gentamicin‐treated group showing degenerative changes in renal glomerulus (black arrow heads) which are surrounded by wide capsular space, degeneration of tubular epithelium (white arrow heads) and accumulation of acidophilic proteinaceous substances inside the lumen of some tubules (black arrows). Stain H&E, Bar = 50 μm. (D) Photomicrograph of renal cortex of gentamicin + *Hyphaene thebaica‐treated group* showing significant amelioration of renal parenchyma with intact renal glomeruli (G) and mild degenerative changes of some renal tubules (arrow heads). Stain H&E, Bar = 50 μm.

Photos shown in Figure 4 show the changes in renal histology in different groups used in this study. The control and *H. thebaica* ‐treated groups show normal structure of renal parenchyma with intact renal corpuscles containing glomeruli (G), which are surrounded by capsular space (arrow heads) in addition to normal proximal (PT) and distal (DT) convoluted tubules. The gentamicin‐injected mice show degenerative changes in renal glomerulus (black arrow heads) which are surrounded by wide capsular space, degeneration of tubular epithelium (white arrow heads) and accumulation of acidophilic proteinaceous substances inside the lumen of some tubules (black arrows) (Figure 4C). In the protective group (GM plus *H. thebaica* ), there is an ameliorative effect of *H. thebaica* against gentamicin, as significant amelioration of renal parenchyma with intact renal glomeruli (G) and mild degenerative changes of some renal tubules (arrow heads, Figure 4D.

### 3.5 Ameliorative Impacts of *Hyphaene thebaica* Treatment on Immunoreactivity of TGF‐β1, NF_k_B, and TNF‐a Against GM‐Induced Renal Toxicity

**FIGURE 5:**
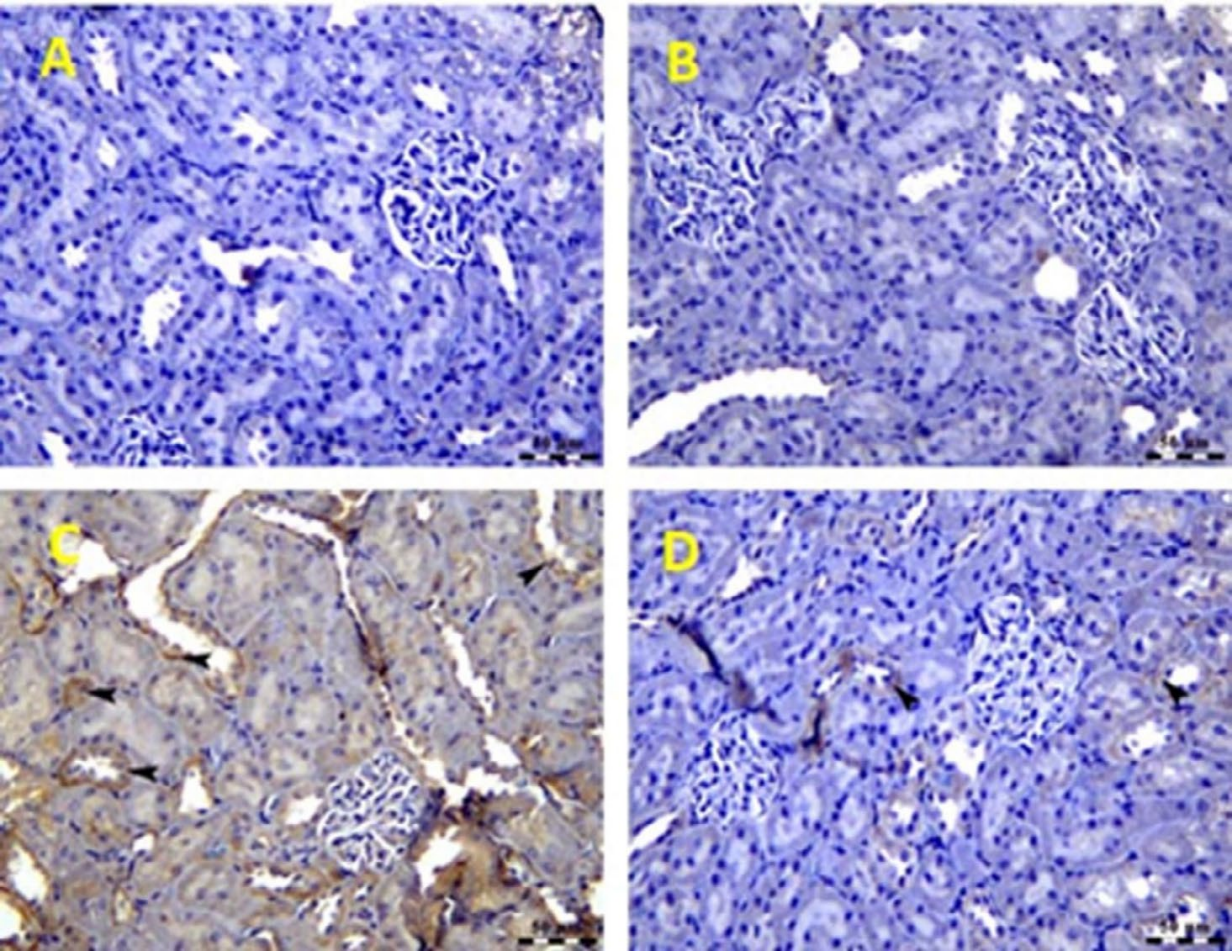
Photomicrograph of liver of the control group (A) and *Hyphaene thebaica* (B) showing negative expression of TGF‐β1 within renal parenchyma. TGF‐β1 IHC, Bar = 50 μm. In (C), the photomicrograph of kidney of the gentamicin‐treated group shows marked expression of TGF‐β1 (arrow heads) in renal tubules. TGF‐β1 IHC, Bar = 50 μm. (D) Photomicrograph of kidney of gentamicin + *Hyphaene thebaica‐treated* group showing weak expression of TGF‐β1 (arrow heads). TGF‐β1 IHC, Bar = 50 μm.

**FIGURE 6:**
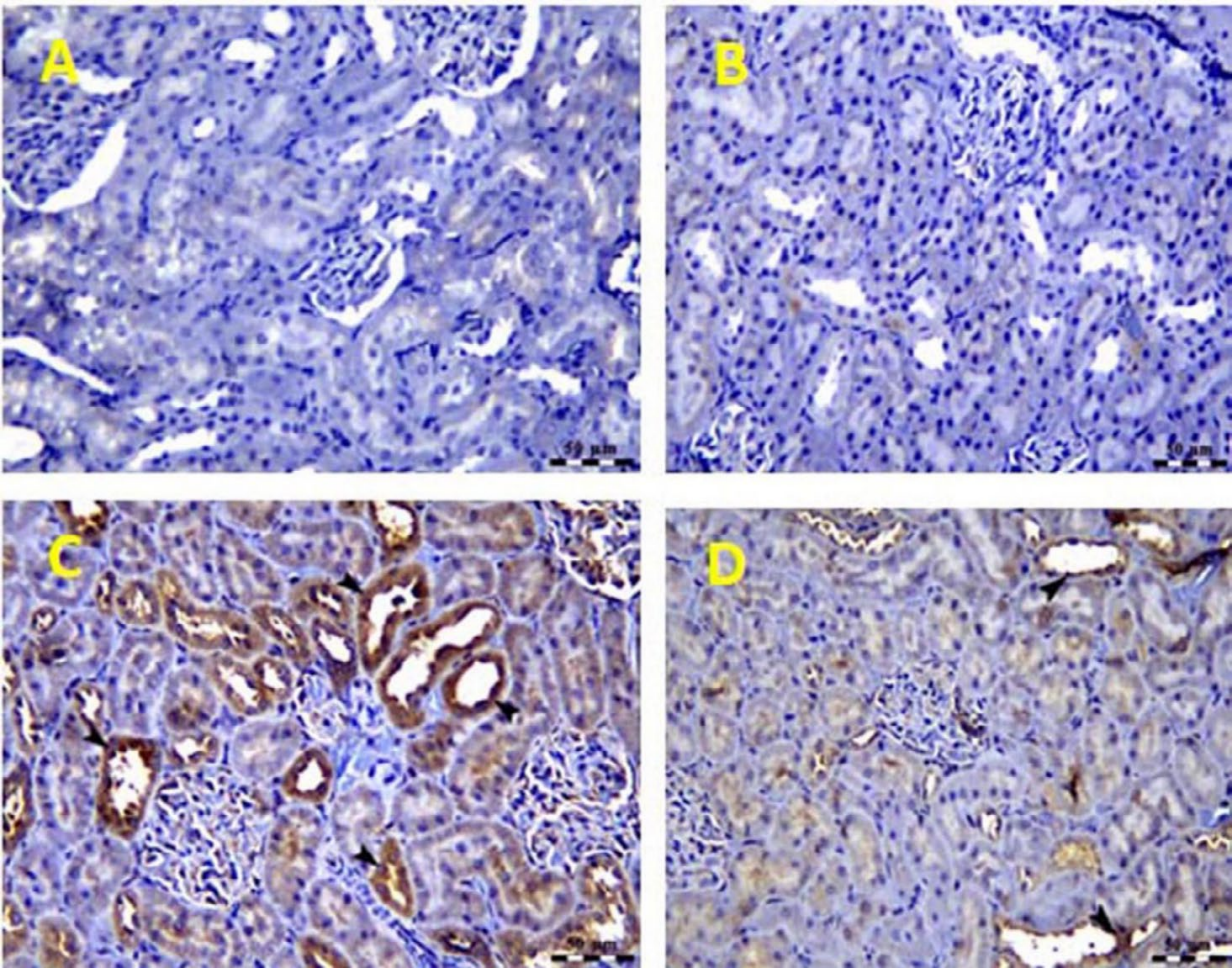
A and B: Photomicrograph of kidney of control and *Hyphaene thebaica* ‐treated groups showing negative expression of NF‐κB in renal parenchyma. NF‐κB IHC, Bar = 50. C: Photomicrograph of kidney of gentamicin‐treated group showing a significant increase of NF‐κB positive renal tubular epithelial cells (arrow heads). NF‐κB IHC, Bar = 50. D: Photomicrograph of kidney of gentamicin + *Hyphaene thebaica* ‐treated group showing a significant decrease in NF‐κB expression within renal tubular epithelium (arrow heads). NF‐κB IHC, Bar = 50 μm.

Figure 5A and B showed that TGF‐β1 immuno‐reactivity was negative in control and *H. thebaica‐receiving* mice (0 immunoreactivity). Gentamycin‐injected mice showed marked expression of TGF‐β1 (arrow heads) in renal tubules (Figure 5C). A clear significant reaction (fourth grade) was reported. These changes were ameliorated when *H. thebaica* was given to gentamycin‐ injected mice (Figure 5D). Moderate significant less reaction (2nd grade) was reported. In parallel, the transcriptional factor NF_k_B showed negative staining in *H. thebaica* and control‐receiving mice (0 immunoreactivity). NF_k_B strong immunoreactivity in renal tubular epithelial cells (arrow heads) was seen in gentamycin‐injected mice (Clear significant reaction (fourth grade) was reported in Figure 6C). This NF_k_B immunoreactivity was decreased when *H. thebaica* was administered to the gentamycin group (moderate significant less reaction (third grade) was reported in Figure 6D).

**FIGURE 7:**
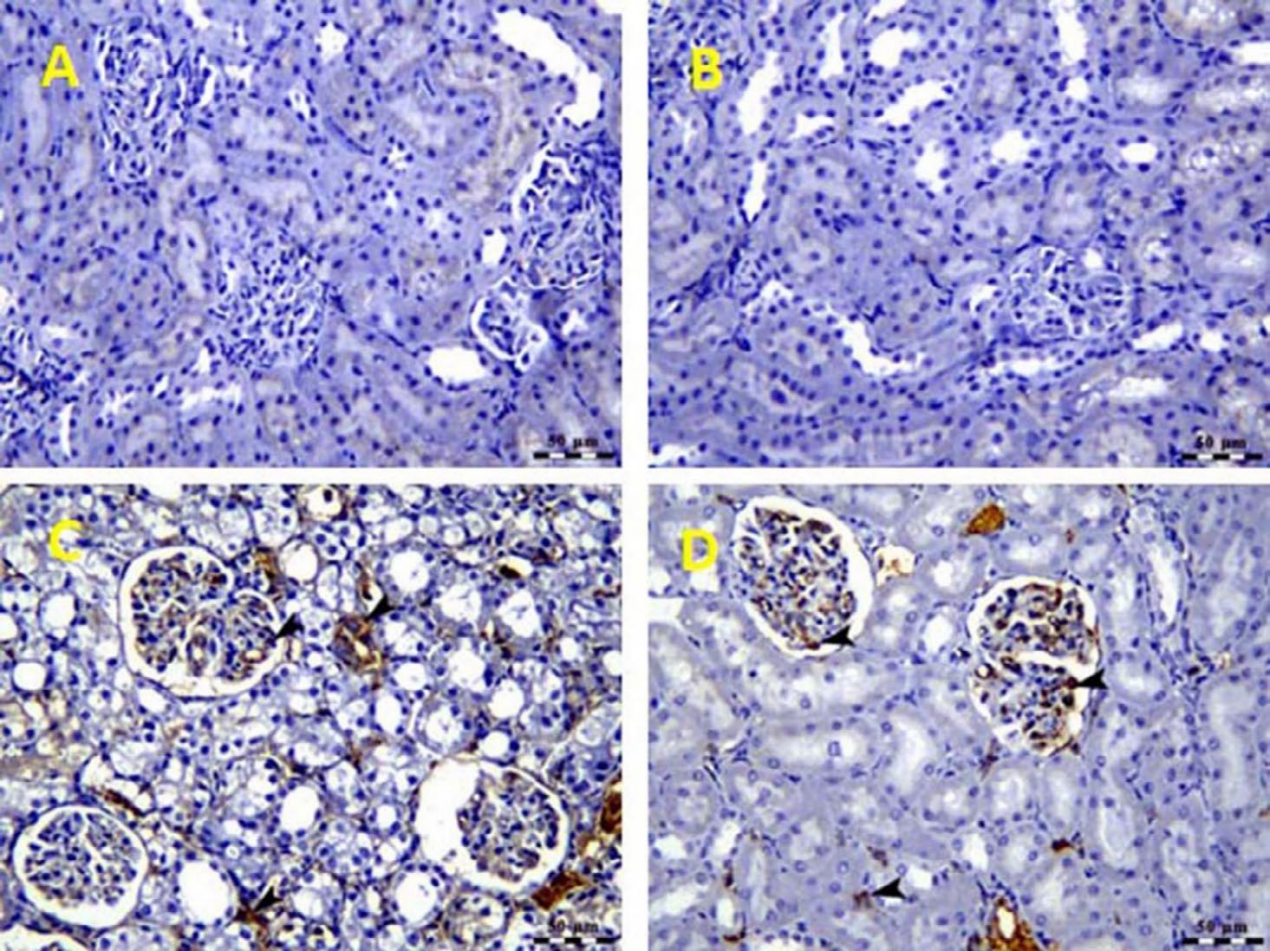
Photomicrograph of the kidney of the control group (A) and *Hyphaene thebaica* (B) groups showing negative expression of TNF‐α. (C) Photomicrograph of the kidney of gentamicin‐treated group showing a significant increase in TNF‐α positive glomerular, tubular, and interstitial tissue (arrow heads). TNF‐α IHC, Bar = 50 μm. (D) Photomicrograph of the kidney of the gentamicin + *Hyphaene thebaica* ‐treated group showing a significant decrease in TNF‐α positive glomerular and interstitial tissue (arrowhead). TNF‐α IHC, Bar = 50 μm.

Finally, the expression of TNF‐α was negative in *H. thebaica* and control‐receiving mice (0 immunoreactivity). The immunoreactivity was high in GM‐injected mice (a clear significant reaction (fourth grade) was reported in Figure 7C) and decreased when *H. thebaica* was given to GM‐intoxicated mice (moderate significant less reaction (second grade) was reported in Figure 7D).

## 4 Discussion

Current findings confirmed the anti‐inflammatory effect of *H. thebaica* against gentamicin‐induced renal dysfunction at cellular levels through different signaling pathways. As known, GM is the most efficient antibiotic against bacteria that have gained resistance to other medications, even though it might affect the kidneys. Gentamicin causes the most prevalent nephrotoxicity in experimental animals (Farombi and Ekor 2006). By activating nuclear factor kappa B, ROS facilitates and initiates inflammation processes (Lopez‐Novoa et al. 2011). The interaction of oxidative stress, organ inflammation, and feedback loops that exacerbate damage and link the mechanisms causing alterations in tubules and glomeruli might be one explanation for the pathophysiology of gentamicin‐induced toxicity (Lopez‐Novoa et al. 2011). An increase in renal enzymes and the presence of histological lesions are common markers for gentamicin nephrotoxic impacts. The pathogenesis of gentamicin‐induced organ damage is mediated through the incidence of inflammation and oxidative stress (Ali et al. 2020; Arjinajarn et al. 2017) as reported in this study.

Increased levels of kidney biomarkers in GM‐injected mice confirmed renal damage, as seen in the present study (Figure 1). Other researchers have found comparable outcomes (Alhyali et al. 2025). Inflammation and alterations in renal physiology are the cause of renal enzymes (2019). Mice receiving *H. thebaica* and GM showed a marked reduction in the levels of biomarkers. This leads us to believe that *H. thebaica* prevented GM‐induced kidney damage by blocking the leakage of kidney markers. It has been found that *H. thebaica* had a nephroprotective effect against kidney damage, confirming the role of *H. thebaica* against organ stress and toxicity (2016). Current renal biomarker findings confirmed the ability of *H. thebaica* to modulate the immune system and reduce inflammation through its ability to regulate the activation of several inflammatory mediators and biomarkers (Ibrahim et al. 2022).

Oxidative stress is characterized by the imbalance between the production and degradation of reactive oxygen species (ROS) or reactive nitrogen species (RNS) (2023). Oxidative stress occurs when the body's capacity to neutralize or repair the destructive effects of ROS occurring on several organs becomes out of balance with the generation of ROS. Numerous documented outcomes were established to combat this oxidative stress and safeguard the health of animals by employing a variety of natural antioxidants derived from plants to restore these damaged organs (2023; Aboubakr, Elmahdy, et al. 2023; Elsayed et al. 2024; Soliman et al. 2024). ROS are molecules whose chemical makeup gives them high reactivity and can come from the metabolism of oxygen or nitrogen. ROS and RNS can be free radicals such as the superoxide radical (O_2_^·‐^), the hydroxyl radical (OH^·^), and the nitric oxide (NO^·^). ROS produce enzymatic reactions within the mitochondria, characterized by the reduction of oxygen through the electron transport chain (Brand 2010). Different cellular processes such as protein phosphorylation, activation of transcription factors, immunity, and apoptosis depend on the cellular concentration of ROS (Rajendran et al. 2014).

In terms of antioxidant and oxidative stress measures, mice treated with gentamycin had significantly higher renal MDA activity in the kidneys of gentamycin‐injected mice compared to control and *H. thebaica*‐administered mice, whereas CAT and GSH levels were significantly decreased. Gentamycin increased ROS production, resulting in lipid peroxidation and subsequently increasing oxidative stress, resulting in the depletion of antioxidant enzymes (Sharma et al. 2022; Yue et al. 2022) and renal apoptosis (Elgazzar et al. 2022). In contrast, GSH, CAT, and MDA were restored toward control levels in the renal tissues of groups pretreated *with H. thebaica*, suggesting antioxidant and protective actions. The antioxidant effect of *H. thebaica* may be attributed to the high content of flavonoids in *H. thebaica* . This was explained by the abundance of flavonoids in *H. thebaica* , which scavenge free radicals or use other mechanisms including metal chelation, lipoxygenase inhibition, and singlet oxygen quenching to prevent lipid oxidation (2003; Abdulazeez et al. 2019). Moreover, *H. thebaica* also has a lot of phenol and flavonoids, which are known to have antioxidant qualities. Phenolic compounds function as antioxidants because they can give off hydrogen molecules, and they can stop lipid oxidation by scavenging radicals, quenching singlet oxygen, chelating metals, and inhibiting lipoxygenase (Huyut et al. 2017). *H. thebaica* acts as an antioxidative stress marker as it enhances the expression of nuclear factor erythroid 2‐related factor 2 (Nrf2), which plays a vital role in regulating the cellular antioxidant (Lestari et al. 2021) and NF_k_B involvement (Alrumaihi 2020). The kidney expressions of pro‐inflammatory cytokines TNF‐alpha, as well as TGF‐β1, NF_k_B, and COX‐2, were significantly reduced after *H. thebaica* administration, as all were upregulated after gentamycin injection and downregulated in the protective group after *H. thebaica* administration.

Results from the histological examination of the gentamycin group showed degenerative changes in the renal glomerulus, which was surrounded by a wide capsular space, accumulation of acidophilic proteinaceous substances inside the lumen of some tubules, and degeneration of tubular epithelium. The results in the protective group showed clear amelioration in the degenerative state of the kidney compared to GM‐injected mice. The protective group showed a decrease in the immunoreactivity of TNF‐α, NF_K_B, and TGF‐β1.

Nuclear factor kB is a transcriptional factor that is mostly associated with different types of inflammation (Wang et al. 2012). It controls mediators of inflammation such as cytokines and chemokines (Lawrence 2009). NFkB promotes the transcription of pro‐inflammatory genes and the inflammatory response following DNA binding (Park et al. 2007). Therefore, inflammation reduction may occur through the NFkB pathway (Sun et al. 2016) and ROS which increase NFkB expression (Herpers et al. 2016). On the other hand, an increase in TNF‐α is responsible for IL‐1β and interferon‐γ secretion (Oku et al. 2002). In the current study, GM‐injection upregulated TNF‐α immunoreactivity (Sakthivel and Guruvayoorappan 2013), increased expression levels of NFkB and TGF‐β1 and COX2 and TNF‐α in the kidney (Hamid et al. 2018).

Oxidative stress activates NFkB, a protein essential for cell division, inflammatory reactions, and programmed cell death (Brasier 2006). In this study, the kidneys of GM‐injected mice showed an elevation of NFkB immunoreactivity, confirming that GM boosted NF‐κB. Tissue damage is associated with the increase in COX‐2 and TGF‐β1 expression. According to this study, NFkB immune‐reactivity was elevated in the kidneys of the experimental animals, indicating that gentamicin treatment increased NF‐κB. Increases in COX‐2 and TGF‐β1 expression were associated with renal injuries (Gao et al. 2010). It was found that *H. thebaica* decreased cyclooxygenase‐2 (COX‐2) upregulation in rats injected with gentamycin due to the presence of flavonoids, coumarins, and saponins in *H. thebaica* (Shalaby and Shata 2013).

Future research should directly examine the stated biological effects in greater detail to emphasize the relevant pharmacodynamic and pharmacokinetic factors. Furthermore, more thorough biological research using mechanistic techniques as well as pre‐clinical and toxicological investigations needs to be assessed to validate the usage of *H. thebaica* formulations.

## 5 Conclusion

**FIGURE 8:**
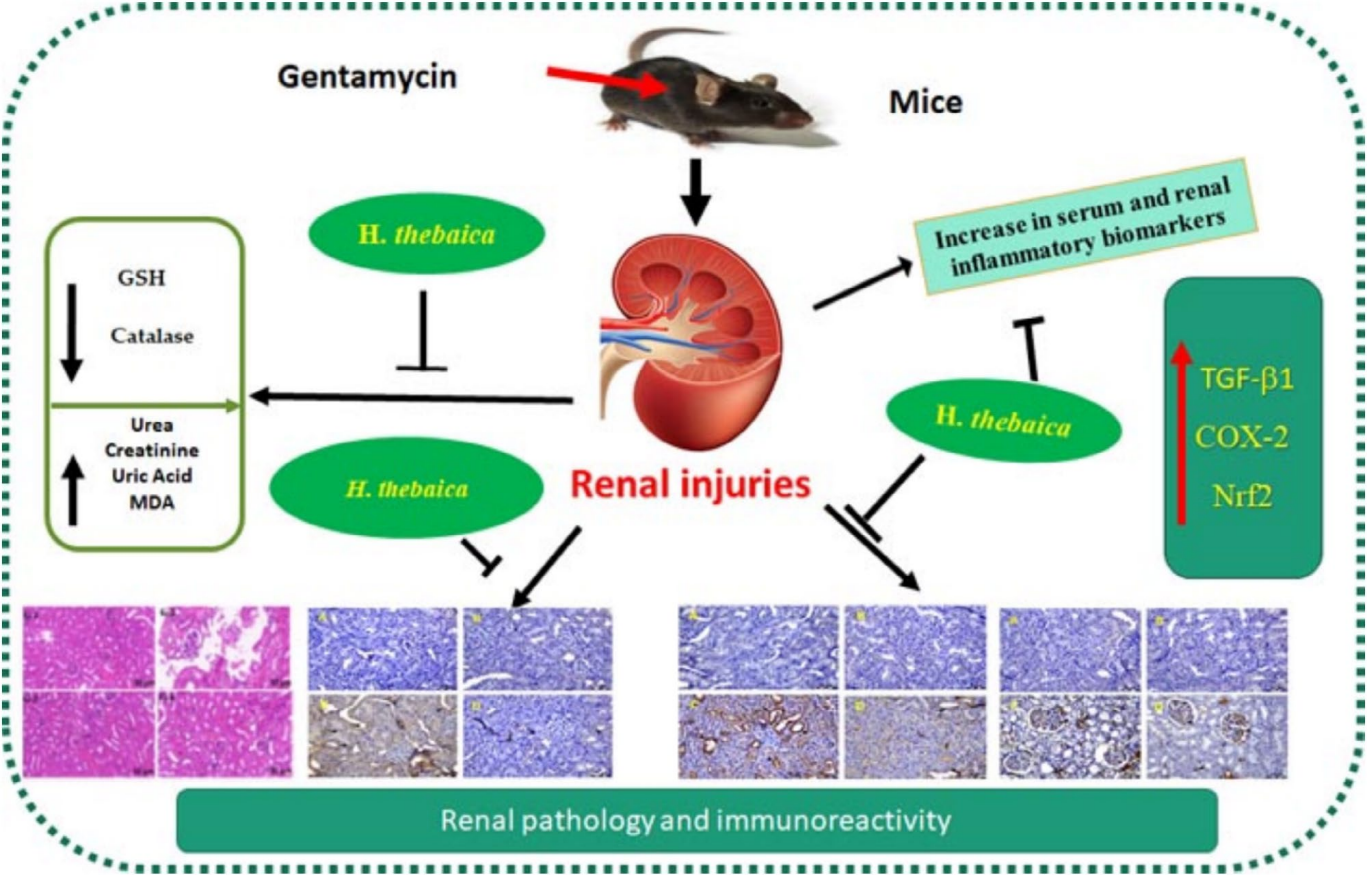
Collective ameliorative impacts of *Hyphaene thebaica* against gentamicin‐induced renal toxicity.

*Hyphaene thebaica* extraction ameliorated nephrotoxic effects of gentamycin through its role as anti‐inflammatory mediators by regulating NFkB, COX‐2, and TGF‐β1 expressions. Moreover, *Hyphaene thebaica* regulated the expression of inflammation‐associated apoptotic genes. *H. thebaica* increased the antioxidative stress biomarker named Nrf2, catalase, and GSH. *H. thebaica* restored the histopathological changes induced by gentamycin. Therefore, using *H. thebaica* as a supplemental nutrient against the toxicity of the kidney is acceptable with limitations to its possible impacts for use in medication. The collective effect of *H. thebaica* is shown in Figure 8.

## Author Contributions

Saed Althobaiti has created, analyzed, written, and submitted this study.

## Conflicts of Interest

The author declares no conflicts of interest.

## Data Availability

Upon request, the data used in this paper can be provided.
